# Molecular diagnosis of sex chromosome mosaics in fetal amniotic cells

**DOI:** 10.1097/MD.0000000000026331

**Published:** 2021-06-25

**Authors:** Zuqian Fan, Xunjin Weng, Zhijian Pan, Qiongying Fan, Ju Long, Guixian Lin, Qian Yang, Lei Sun

**Affiliations:** a Department of Clinical Laboratory, Qinzhou Maternal and Child Health Hospital, Guangxi; b Qinzhou Key Laboratory of Molecular and Cell Biology on Endemic Diseases, Qinzhou, Guangxi; c Laboratory of Medical Genetics, Qinzhou Maternal and Child Health Hospital, Guangxi; d Laboratory Animal Center, Xi’an Jiaotong University Health Science Center, Xi’an, Shaanxi, PR China.

**Keywords:** chromosome, FISH, karyotype analysis, mosaic, SD-QF-PCR

## Abstract

Mosaicism can be observed in karyotype analyses of amniotic fluid cells. Differentiating between true mosaicism and pseudomosaicism and determining mosaic proportions can help avoid misjudgments by doctors and effectively reduce mental and physical harm to pregnant women. However, the detection of mosaicism and mosaic proportions via karyotype analysis and fluorescence in situ hybridization (FISH) is extremely complex. We have developed a novel approach, “segmental duplication quantitative fluorescent PCR” (SD-QF-PCR), to detect mosaicism and mosaic proportions.

In this study, twenty control samples and fourteen mosaic samples were tested by first-line karyotype analysis; by second-line karyotype analysis, SD-QF-PCR and FISH were used to reassess fetal sex chromosome mosaicism and mosaic proportions.

Detection of the 20 control samples by second-line karyotype analysis via FISH and SD-QF-PCR showed normal and consistent results. Among the 14 mosaic samples, the numbers of samples showing true mosaicism and pseudomosaicism detected by the three methods were 6 and 8, respectively.

Our study demonstrates that SD-QF-PCR can be used as a complementary method to traditional cytogenetic analysis of amniotic fluid mosaics and has clinical application value.

## 1 Introduction

Fetal chromosomal abnormalities can be detected by amniotic fluid cell culture and karyotype analysis during the second trimester of pregnancy. Because of its detection scope and reliability, karyotype analysis has become the gold standard for detecting fetal chromosomal abnormalities.^[1]^ Karyotype analysis is also one of the most widely used prenatal diagnostic methods. However, mosaicism is often detected via karyotype analyses due to the long cultivation cycle of amniotic fluid cells and the complicated preparation process involved in such analyses.

Mosaicism detected in prenatal diagnoses of amniotic fluid is classified as true fetal mosaicism (TFM) or pseudomosaicism. TFM is mainly caused by mitotic nondisjunction or chromosomal loss; it may also be caused by the absence of a chromosome in an individual daughter cell and its offspring due to chromatid retention in the anaphase stage of mitosis.^[2]^ Pseudomosaicism, which is a culture artifact, is caused by maternal cell contamination or aberrations in exfoliated fetal cells during cultivation or the chromosome production process.^[3]^ TFM can severely affect fetal growth and mental development,^[4,5]^ whereas pseudomosaicism leads to unnecessary prenatal interventions that cause significant mental and physical harm to pregnant women. Therefore, in current prenatal diagnoses, it is extremely important to accurately determine whether an observed mosaic represents TFM or pseudomosaicism to ensure that appropriate guidance and genetic counseling are provided.

If fetal mosaicism is detected in the amniotic fluid using karyotype analysis, recommendations suggest careful treatment rather than termination of the pregnancy.^[6,7]^ Test results should be further confirmed using second-line karyotype analysis, cord blood or other detection techniques such as FISH,^[8]^ and Doppler ultrasound should be utilized for systematic examination. If necessary, tests on multiple tissues should be conducted after the child is born, and postnatal follow-up should be enhanced.

With the development of molecular biology techniques, a number of diagnostic approaches have been utilized to diagnose fetal chromosome aneuploidy, such as FISH^[9,10]^ short tandem repeat analysis (STR-QF-PCR)^[11]^ and chromosomal microarray techniques.^[12]^ All of these diagnostic techniques have the advantage of rapid diagnosis. However, STR-QF-PCR cannot be used to assess mosaic proportions; thus, STR-QF-PCR cannot effectively reveal the extent of chromosome aberration during the cell culture process. FISH can effectively detect mosaic proportions but has the main drawbacks of being overly cumbersome and complex and requiring experienced technical staff. Chromosomal microarray technology can effectively detect mosaicism, but the cost of the instruments and reagents is extremely high, leading to a low level of acceptance of this approach in the developing world.

In our previous study, we developed a new detection method, “segmental duplication quantitative fluorescent PCR” (SD-QF-PCR),^[13]^ that can be used for the rapid diagnosis of aneuploidy. Upon our further study, we found that this quantitative approach was extremely accurate and stable and was therefore well suited for the detection and analysis of mosaicism and its proportions. Therefore, in this paper, we used the SD-QF-PCR technique to analyze amniotic fluid cell samples for mosaicism and compared this technique with FISH and traditional karyotyping, this approach allowed us to deeply investigate the clinical applicability and value of SD-QF-PCR for diagnosing chromosomal abnormalities.

## 2 Materials and methods

### 2.1 Ethics statement

This study was conducted in accordance with the principles expressed in the Declaration of Helsinki. The study protocol was approved by the Research Ethics Committee of Qinzhou Maternal and Child Health Hospital. Written informed consent was obtained from each participant for the collection of samples and subsequent analyses.

### 2.2 Samples

Twenty cases involving amniotic fluid samples with normal karyotypes and fourteen cases involving amniotic fluid samples with mosaicism were selected for this study. All 34 samples had undergone first-line amniotic fluid cell culture and karyotype analysis. Second-line amniotic fluid karyotype analysis was performed for validation.

### 2.3 FISH detection of original amniotic fluid cells

X- and Y-chromosome centromere probes (GPMEDICAL) were used for FISH analyses of original amniotic fluid samples in all 34 included cases. In accordance with the manufacturer's instructions, 100 total cells were randomly counted for each sample, the types of sex chromosomes were determined based on the fluorescence signals of the X and Y chromosomes in cells, and mosaicism and mosaic proportions were determined using these fluorescence signals.

### 2.4 Segmental duplications and primers

Segmental duplications are nearly identical DNA segments^[14]^ and can be obtained from NCBI (http://www.ncbi.org/). The segmental duplications that can be used for QF-PCR detection and have diagnostic value refer to two similar sequences. The bases at both ends of similar sequences are identical for primer design, while the number of bases in the middle is different for copy number detection. Meanwhile, one of the similar sequences must be located on the target chromosome, and the other similar sequences, with different sizes, must be located on a chromosome other than the target chromosome.

### 2.5 SD-QF-PCR detection of original amniotic fluid samples

Original amniotic fluid samples in all 34 cases were assessed via SD-QF-PCR. PCR amplification was performed using the Gene Amp PCR System 9700 (Applied Biosystems) in a total reaction volume of 25 μL containing 1× Reaction Master Mix (Tiangen Biotech), 10 ng genomic DNA and 0.5 μmol/L of each forward and reverse primer. The reaction mixture was preheated at 95°C for 3 min, followed by 28 cycles of 30 s at 95°C, 30 s at 60°C, and 30 s at 72°C and a final extension step at 72°C for 10 min. Approximately 1 μL of the PCR product was mixed with 24 μL of formamide and 1 μL of the GeneScan 500 Rox size standard (Applied Biosystems). The mixture was performed using a POP4 gel (Applied Biosystems) on the ABI 3130xl Genetic Analyzer (Applied Biosystems). GeneMapper ID Software v3.2 (Applied Biosystems) was used for the data analysis.

### 2.6 Results analysis

The ratio of the segmental duplications in two different chromosomes detected by SD-QF-PCR was converted to the mosaic proportion of different karyotypes, and this mosaic proportion was compared with that detected by first-line amniotic fluid karyotype analysis, with the second-line karyotype analysis and FISH analysis used to verify the accuracy and feasibility of the SD-QF-PCR method.

## 3 Results

### 3.1 Segmental duplications and primers

In this SD-QF-PCR experiment, two segmental duplications were used to detect numbers of sex chromosomes. Fragments from segmental duplications between chromosome 3 and chromosome X were used to calculate the copy number for the X chromosome, and segmental duplications between chromosome X and chromosome Y were used to detect the copy numbers for the X and Y chromosomes.

**Figure 1:**
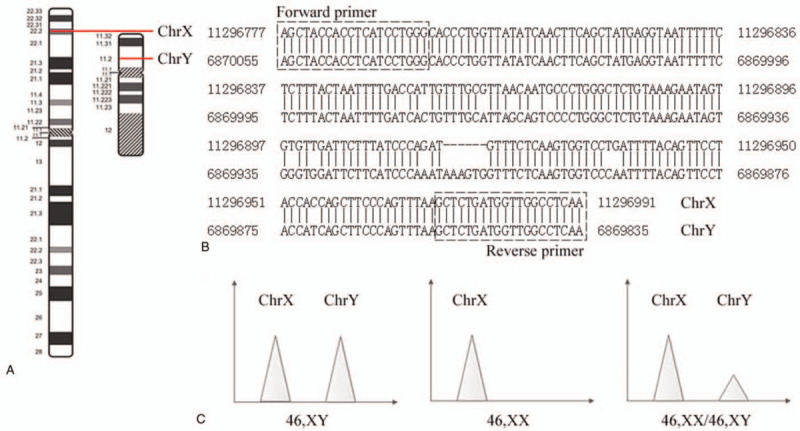
The segmental duplication of chromosomes X and Y.

Figure 1 shows the segmental duplications of chromosome X and Y, primer design and detection principle. Figure 1A shows the positions of the segmental duplications on the chromosome X and Y, Figure 1B shows the bases and primers of the segmental duplications (two similar sequences), and Figure 1C shows the SD-QF-PCR pattern of the normal female, normal male and mosaic.

### 3.2 Conversion of the ratio to the mosaic proportion

**Figure 2:**
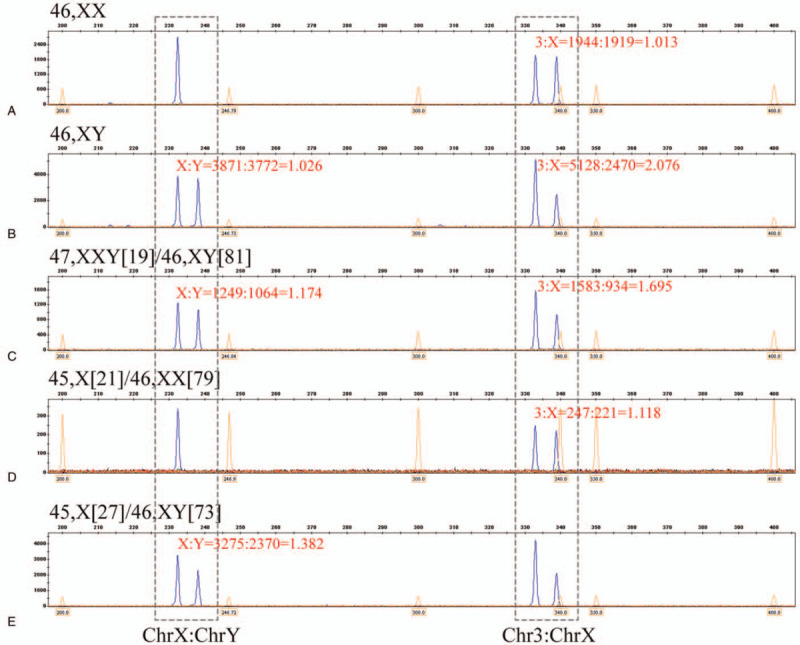
Capillary electrophoresis analysis for SD-QF-PCR.

The result of SD-QF-PCR detection is the fluorescence ratio between the segmental duplications (also the ratio between the two chromosomes). We can only compare the result with other methods after converting the fluorescence ratio into the mosaic proportion. The calculation formula for conversion is as follows: Taking X:Y=1.174 (Fig. 2C) in the 47, XXY[19]/46, XY[81] mosaic sample as an example, we assume that a total of 100 karyotypes are analyzed, of which the number of 46, XY karyotypes is α and that of 47, XXY karyotypes is (100-α). First, the number of X chromosomes in 100 karyotypes is calculated. Since there is only one X chromosome in each 46, XY karyotype, the number of X chromosomes is α in this karyotype. Furthermore, there are two X chromosomes in each 47, XXY karyotype; thus, the number of X chromosomes is 2∗(100-α) in this karyotype. Therefore, the total number of X chromosomes is [α+2∗(100-α)]. Second, the number of Y chromosomes in 100 karyotypes is calculated. Since there is only one Y chromosome in each 46, XY karyotype, the number of Y chromosomes is α in this karyotype. Furthermore, there is also only one Y chromosome in each 47, XXY karyotype; thus, the number of Y chromosomes is (100-α) in this karyotype. Therefore, the total number of Y chromosomes is [α+(100-α)]. Finally, according to the SD-QF-PCR results, the ratio of segmental duplications is X:Y=1.174, and the formula can be written as follows: X:Y = [α+2∗(100-α)]/[α+(100-α)] = 1.174. Hence, we get α=82.6≈83. Therefore, the mosaic proportions of different karyotypes can be obtained: [47,XXY]:[46,XY] = (100-83):83 = 17:83. By converting the ratio of segmental duplications to the mosaic proportions of different karyotypes, the results can be compared easily with those of other methods. In addition, other segmental duplication ratios can be converted using this method.

### 3.3 First-line amniotic fluid karyotype analysis

**Figure 3:**
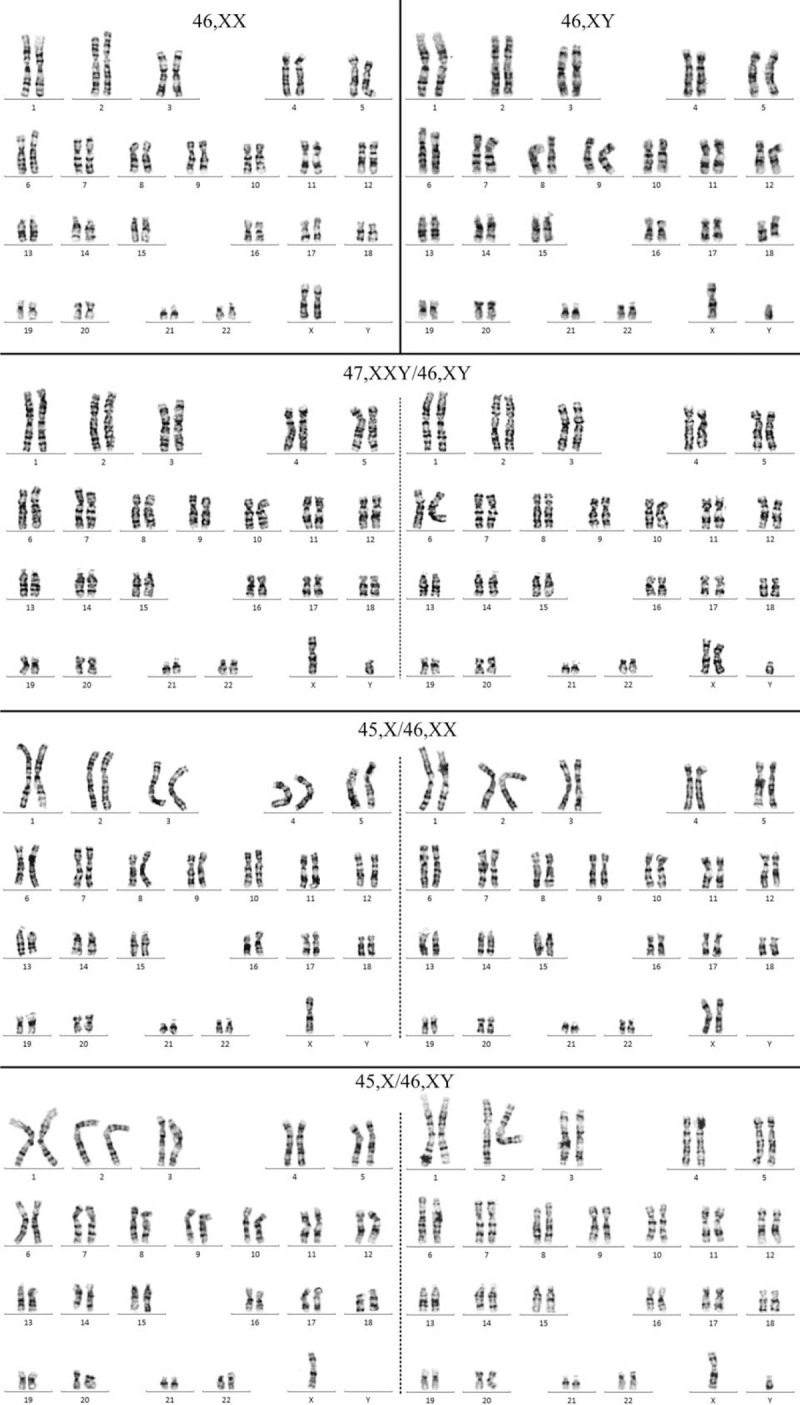
Karyotypes of the normal and mosaic chromosomes.

Thirty-four clinical amniotic fluid samples were detected using karyotype analysis, of which 20 control samples were obtained from individuals with the normal karyotype 46, XX or 46, XY. One sample was obtained from an individual with the karyotype 47, XXY/46, XY; 8 samples were obtained from individuals with the karyotype 45, X/46, XX; and 5 samples were obtained from individuals with the karyotype 45, X/46, XY. The partial karyotypes of the normal and mosaic chromosomes are shown in Figure 3, and the mosaic proportions are presented in Table 1.

**Table 1:** Mosaic proportions of the samples determined using different detection methods.

		Karyotype analysis			SD-QF-PCR	
No.	Karyotype from First-line analysis	First- line analysis	Second- line analysis	FISH	ChrX: ChrY	Chr3: ChrX
1	47, XXY/46, XY	25:75	32:68	19:81	17:83 (1.174)	18:82 (1.695)
2	45, X/46, XX	38:62	15:85	21:79	–	21:79 (1.118)
3	45, X/46, XX	19:81	29:71	14:86	–	17:83 (1.093)
4	45, X/46, XX	42:58	37:63	48:52	–	47:53 (1.305)
5	45, X/46, XX	5:95	0:100	1:99	–	2:98 (1.012)
6	45, X/46, XX	18:82	2:98	0:100	–	2:98 (1.009)
7	45, X/46, XX	7:93	0:100	0:100	–	3:97 (1.014)
8	45, X/46, XX	11:88	1:99	0:100	–	3:97 (1.015)
9	45, X/46, XX	8:92	1:99	0:100	–	1:99 (1.007)
10	45, X/46, XY	22:78	33:67	27:73	28:72 (1.382)	–
11	45, X/46, XY	21:79	7:93	10:90	17:83 (1.208)	–
12	45, X/46, XY	13:87	1:99	0:100	1:99 (1.015)	–
13	45, X/46, XY	7:83	0:100	1:99	1:99 (1.016)	–
14	45, X/46, XY	27:73	1:99	0:100	2:98 (1.025)	–

### 3.4 Second-line amniotic fluid karyotype analysis

All 34 samples were revalidated by second-line amniotic fluid karyotype analysis. The results of the 20 control samples were the same as those of the first-line analysis. In 14 suspected mosaic samples, the results of second-line amniotic fluid karyotype analysis indicated that 1 sample from an individual with the karyotype 47, XXY/46, XY; 3 samples from individuals with the karyotype 45, X/46, XX (5 samples with 46, XX); and 2 samples from individuals with the karyotype 45, X/46, XY (3 samples with 46, XY). All of the mosaic proportions are presented in Table 1.

### 3.5 FISH detection of original amniotic fluid cells

FISH probes were used to assess 14 amniotic fluid samples, of which 6 amniotic fluid samples were determined to be mosaics, and 8 amniotic fluid samples were of normal sex chromosome karyotype. The results were consistent with the results of the second-line amniotic fluid karyotype analysis. All of the mosaic proportions are presented in Table 1.

### 3.6 SD-QF-PCR detection of original amniotic fluid cells

In normal female samples, the chromosome 3: chromosome X ratio was 1944: 1919 = 1.013, and chromosome Y was not amplified (Fig. 2A). In normal male samples, the chromosome 3: chromosome X ratio was 5128: 2470 = 2.076, and the chromosome X: chromosome Y ratio was 3871: 3772 = 1.026 (Fig. 2B).

In the SD-QF-PCR detection, 6 amniotic fluid samples were determined to be mosaics, and 8 amniotic fluid samples were of normal sex chromosome karyotype. The segmental duplication ratios and the karyotype proportions of the 14 amniotic fluid samples are presented in Table 1. In addition, Figure 2A&B shows the segmental duplications fluorescence ratios for the normal karyotype, whereas Figure 2C–E shows the different mosaic proportions for karyotypes with different fluorescence ratios.

## 4 Discussion

According to our results and those of other scholars, the main causes of mosaicism is maternal cell contamination or chromosomal aberrations produced during the in vitro cultivation of fetal exfoliated cells; thus, pseudomosaicism occurs more frequently than TFM.^[15]^ Therefore, when a mosaic karyotype is observed in the amniotic fluid, the pregnancy should not be terminated immediately; instead, the karyotype results should be further validated via second-line amniotic fluid cell culture and karyotype analysis or cord blood karyotype analysis, or even FISH. If these additional assessments indicate TFM, tissues from the aborted fetus or the postnatal neonate can be evaluated to reach a final diagnosis.^[16,17]^

At present, second-line amniotic fluid karyotype analysis or cord blood karyotype analysis is the most common method for verifying first-line amniotic fluid results; however, second-line karyotype analysis also brings the possibility of pseudomosaicism. Therefore, if mosaicism occurs during amniotic fluid karyotyping, the best follow-up approach is to use the original amniotic fluid cells to determine whether a mosaic karyotype exists and thereby accurately differentiate between TFM and pseudomosaicism.^[18]^ Among the current techniques, FISH^[9,10]^ is an accurate means of testing for mosaicism. In this study, we developed a simpler and faster SD-QF-PCR approach to detect mosaicism and mosaic proportions. This technique can not only be used for the detection of amniotic fluid cells, but if it is applied to tissues from the chorionic villus, the aborted fetus or the postnatal neonate that cannot be cultured, this technique may be more advantageous than FISH or karyotyping.

The results of the karyotype analysis, FISH and SD-QF-PCR are shown in Table 1. In the first-line karyotype analysis, we found 14 cases of mosaic karyotypes. There were more than 3 abnormal karyotypes in the mosaic sample. Therefore, we thought it might be a mosaicism and proceeded to the next step immediately. In the second-line karyotype analysis, FISH and SD-QF-PCR were used to reassess the results of the first-line amniotic fluid karyotype analysis. FISH results are the gold standard for mosaic detection. Therefore, we compared the results of the second-line karyotype analysis and SD-QF-PCR with those of the FISH analysis to judge the accuracy of the karyotyping and SD-QF-PCR technology.

The results of 20 control samples from 3 methods indicated that all samples had normal sex chromosomes. In 14 amniotic fluid cell mosaic samples, the results of the second-line amniotic fluid karyotype analysis, FISH and SD-QF-PCR indicated that 6 samples had the TFM karyotype and 8 samples showed pseudomosaicism. Pseudomosaicism was found in 45, X/46, XX and 45, X/46, XY karyotypes, which is caused by aberrations in exfoliated fetal cells during cultivation or the chromosome production process. The results showed that all the three techniques can be used to identify TFM and pseudomosaicism.

Although the three techniques can distinguish TFM and pseudomosaicism, the mosaic proportion detected by the three techniques is different in TFM. Since FISH technology is used to detect uncultured amniotic fluid cells, this technology can well reflect the mosaic proportion of original amniotic fluid cells. The karyotype analysis requires cell culture, and the attachment and growth of different cell types in the mosaic sample are different. Therefore, the results of the first-line karyotype analysis and the second-line karyotype analysis will be quite different (such as No. 2), which cannot accurately reflect the mosaic proportion of the original amniotic fluid cells. The SD-QF-PCR technique is similar to the FISH technique. Both techniques are used to detect uncultured amniotic fluid cells. The results in Table 1 show that the results of SD-QF-PCR are very close to the FISH results, and the difference is significantly smaller than the difference between karyotype analysis and FISH. The SD-QF-PCR result is more accurate than the karyotype analysis to determine the mosaic proportion. Therefore, the SD-QF-PCR technology can be used not only to judge mosaicism but also to determine the mosaic proportion.

The SD-QF-PCR technology has very good development prospects, but there are still many areas that need further research. For example, although sex chromosome mosaicism occurs most, other chromosomes also have mosaicism. Therefore, it is necessary to further develop the detection system for other chromosomes. In addition, because the sample size is a little small, we cannot clarify the detection limit for the method. However, according to our current research, mosaicism is completely detectable for proportions above 10%. We will further improve this in the follow-up.

## 5 Conclusions

In conclusion, our study demonstrates that similar to FISH, SD-QF-PCR can be used as a complementary method to traditional cytogenetic analyses in the evaluation of amniotic fluid mosaics.

## Author contributions

**Conceptualization:** Zuqian Fan, Qian Yang, Lei Sun.

**Data curation:** Zuqian Fan, Ju Long.

**Funding acquisition:** Zhijian Pan, Lei Sun.

**Investigation:** Zhijian Pan.

**Methodology:** Ju Long.

**Project administration:** Xunjin Weng.

**Resources:** Xunjin Weng, Qian Yang.

**Software:** Qiongying Fan.

**Supervision:** Qiongying Fan.

**Validation:** Guixian Lin.

**Writing – original draft:** Lei Sun.

**Writing – review & editing:** Lei Sun.
